# Response to ‘T-helper 17 cell cytokines and interferon type I: partners in crime in systemic lupus erythematosus?’ – Authors’ reply

**DOI:** 10.1186/ar4578

**Published:** 2014-06-12

**Authors:** Zana Brkic, Odilia BJ Corneth, Marjan A Versnel, Erik Lubberts

**Affiliations:** 1 Department of Immunology, Erasmus MC, University Medical Center, Wytemaweg 80, 3015 CN Rotterdam, The Netherlands; 2 Department of Rheumatology, Erasmus MC, University Medical Center, Wytemaweg 80, 3015 CN Rotterdam, The Netherlands

## 

We would like to reply to the letter by Dolff and colleagues [1] regarding our article in a recent issue of *Arthritis Research & Therapy*[2]. We thank the authors for their interest in our work and the critical reading of our article. The aim of our study was to investigate a possible association between the IFN type I signature and memory T helper 17 (Th17) cells and their cytokines in systemic lupus erythematosus (SLE). Since CCR6 can be expressed by regulatory T (Treg) cells, we have excluded CD25^high^ cells to discriminate between CD4^+^CD45RO^+^CCR6^+^CD25^−^ (primary Th17 cells) and Treg cells. Concerning the observation on co-expression of IL-17A with IFN-γ, we would like to remark that IFN-γ is an IFN type II, and not an IFN type I, cytokine and does not bind to the IFN type I receptor. Detection of IFN type I activity is hampered by the difficulty to assess the different subtypes of this cytokine. Therefore, analysis of IFN type I-induced gene expression in RNA from peripheral blood cells, the so-called IFN type I signature, is used as a measure for IFN type I activity. Although we fully agree with the authors that the relationship between Th cells and IFN type I deserves further study, their remark on the ‘genetic’ signature is confusing and probably refers to the IFN type I-induced gene expression signature, which is detected at the RNA level.

The relationship between CD25^+^ Tregs and IFN type I is certainly of interest for further study as these Tregs are carrying the IFN type I receptor and thus respond to increased systemic IFN type I activity in SLE. Also, follow-up studies with a focus on adaptive and innate cells producing IL-17, including the relation with IL-21 and IL-22 and plasticity, will be of interest [3,4]. In our article, we presented data supporting a potential co-activity between IFN type I and CD4^+^memory CCR6^+^ Th17 cytokines. Further studies are needed to confirm this co-activity with a focus on revealing the mechanism of this dangerous link in SLE.

### Abbreviations

IFN: Interferon; IL: Interleukin; SLE: Systemic lupus erythematosus; Th17: T helper 17; Treg: Regulatory T.

### Competing interests

The authors declare that they have no competing interests.
